# The effect of 25-hydroxyvitamin D levels on QT interval duration and dispersion in type 2 diabetic patients

**DOI:** 10.3325/cmj.2015.56.525

**Published:** 2015-12

**Authors:** Demet Ozgil Yetkin, Belgin Kucukkaya, Mehtap Turhan, Merve Oren

**Affiliations:** 1 Department of Endocrinology, Kolan International Hospital, Istanbul, Turkey; 2 Department of Internal Medicine, Bayindir Hospital, Istanbul, Turkey; 3 Department of Biochemistry, Bayindir Hospital, Istanbul, Turkey; 4 Department of Public Health, Istanbul University, Istanbul Medical Faculty, Istanbul, Turkey

## Abstract

**Aim:**

To assess the relationship between corrected QT (QTc) interval and vitamin 25-hydroxyvitamin D levels (25-OHD) deficiency in type 2 diabetic patients.

**Methods:**

The study included 253 patients with type 2 diabetes and 170 age-matched controls treated between October and December 2013. QTc duration and QTc dispersion were measured on ECG recordings and 25-OHD, calcium, phosphorus, and blood glucose levels were determined.

**Results:**

Patients with diabetes had significantly longer QTc duration and QTc dispersion than controls (*P* < 0.001 and *P* < 0.001 respectively). Diabetic patients with prolonged QTc duration were older and had longer diabetes duration and higher HbA1c levels than patients with normal QTc interval. They significantly more frequently had 25-OHD deficiency (*P* < 0.001), but had similar calcium and phosphorus levels. Diabetic patients with prolonged QTc dispersion were of similar age and had similar diabetes duration and HbA1c levels as patients with normal QTc dispersion. They significantly more frequently had 25-OHD deficiency (*P* = 0.010), but had similar calcium and phosphorus levels.

**Conclusion:**

This study showed prolonged QTc duration and QTc dispersion in patients with type 2 diabetes, especially those with 25-OHD deficiency.

Worldwide prevalence of type 2 diabetes mellitus (T2DM) is 7.7%, and is expected to further increase. The principal cause of death in patients with T2DM are cardiovascular diseases, with the annual mortality of 5.4% (1). Cardiac mortality and morbidity rate can be reduced by disease detection at the time when overt cardiac disease is still not present. This requires novel screening strategies, one of which is QT interval analysis. QT abnormalities can predict cardiac death in several diseases, including chronic heart failure, systemic hypertension, and peripheral vascular disease (2). In diabetic patients QT abnormalities can be detected at diagnosis and are better predictors of cardiac death than autonomic function tests (3). Moreover, in diabetic patients increased QT dispersion was shown to predict cardiovascular mortality (4).

Calcium-phosphorus metabolism disorders and 25 hydroxyvitamin D (25-OHD) deficiency are known risk factors for cardiovascular events (5-7). Also, disturbances that lead to decreased 25-OHD levels have been shown to result in structural and ionic channel remodeling, which may increase the susceptibility to cardiac arrhythmias. In addition, they induce cardiac hypertrophy and fibrosis, which are known risk factors for sudden cardiac death (7,8).

Altered 25-OHD and calcium homeostasis may also play a role in the development of T2DM. Vitamin D has been shown to affect the synthesis and secretion of insulin (9) and predict the increased risk of all-cause and cardiovascular mortality in T2DM patients independent of conventional risk factors (10). However, there are limited data on the relationship between 25-OHD and QT parameters in diabetic patients. The aim of this study was to investigate the relationship between the 25-OHD deficiency and the corrected QT (QTc) interval duration and dispersion in patients with diabetes.

## Patients and methods

The study included 253 (129 women) consecutive patients with type 2 diabetes (mean age of 59.7 years and standard deviation [SD], 6.5), followed and treated at our outpatient clinic between October and December 2013. The control group included 170 (87 women) healthy volunteers (mean age of 59.1 years and SD 5.5) who attended their routine check-ups at our clinic.

Vitamin D deficiency was defined as 25-OHD concentration of less than 30 mg/mL (11). Patients who were treated with vitamin D and calcium within one year before the study were not included. We determined participants’ characteristics including age, duration of diabetes, and hypertension, medications, and presence of complications including retinopathy, nephropathy, and peripheral neuropathy. Retinopathy was diagnosed by an ophthalmologist and microalbuminuria was defined as urine albumin to creatinine ratio between 30 and 300 mg/g. Peripheral neuropathy was diagnosed on the basis of neuropathic symptoms and signs or objectively abnormal results, including insensitivity to a 10 g monofilament, abnormal vibration perception threshold, measured using a biothensiometer, and a lack of any other significant disease (12).

Patients with known coronary artery disease, defined as the history of myocardial infarction (MI), coronary artery bypass grafting surgery, percutaneous coronary intervention, and the presence of coronary heart disease, as documented by coronary angiography, were not included in the study. Patients with chronic renal failure, macroalbuminuria, and acute illness 24 hours prior testing were also not included. None of the patients were receiving drugs that could lengthen the QT interval, such as class IA, IC, or III antiarrhtymic drugs. Patients with complete bundle branch block, atrial fibrillation, second or third degree atrio-ventricular block, or less than six measurable leads in the ECG and patients with insulin treatment were excluded. All patients were taking oral hypoglycemic agents. The study was approved by the ethics committee of the Bayindir Hospital and all patients provided written informed consent.

ECG was performed with the patients lying comfortably in supine position. Digital 12-lead ECG recordings were analyzed at an ECG paper speed of 25 mm/s using a M1171A Pagewritter 200 (Philips, Andover, MA, USA). The QT intervals for each lead were measured manually from the first deflection of the QRS complex to the point of the T wave offset, defined by the return of the terminal T wave to the isoelectric TP baseline. Each QT interval was corrected for patient’s heart rate using Bazett’s formula: QTc = QT√RR (ms) (13), where QTc >440 ms was considered prolonged. QTc dispersion was calculated using the difference between the maximum and the minimum QTc in any lead. QT dispersion >80 ms was considered prolonged.

Blood samples were collected after an overnight fasting from the medial cubital vein. Serum calcium and phosphorus were measured with photometric system (Roche Cobas C501, Roche Diagnostics, Indianapolis, IN, USA). 25-OHD and hemoglobin A1c (HbA1c) were measured by turbidimetric inhibition immunoassay (Roche Cobas C51, Roche Diagnostics).

Data were analyzed by IBM Statistics for Windows, version 21.0 8 (IBM Corp., Armonk, NY, USA). Normality of the distribution was tested with Kolmogorov-Smirnov test. Continuous data were expressed as mean±SD and median with interquartile ranges when appropriate. *t* test and Mann-Whitney-U test, depending on the normality of distribution, were used to test the differences between the study groups. χ^2^ test was used for the categorical variables. Logistic regression analyses (stepwise, forward) were used to estimate odds ratios (ORs) and the 95% confidence intervals (95% CIs). In the regression model, the QTc prolongation was entered as dependent variable, and age, duration of diabetes and vitamin D deficiency as independent variables. Post hoc analysis was performed, and the power of the study with given sample size was 0.91 (two means *t* test). A two-tailed *P* value of 0.050 was considered significant.

## Results

Mean duration of diabetes was 9.6 ± 6.6 years. Mean HbA1c level was 7.1 ± 1.1% (54 mmol/mol) and median fasting blood glucose level was 133 (IQR 111-164) mg/dL. Retinopathy was present in 85 (33.5%), neuropathy in 73 (28.8%), microalbuminuria in 75 (26.9%), and hypertension in 108 patients (42.6%) (Table 1).

**Table 1:** Demographic and laboratory data of patients and controls*

	Diabetes mellitus patients (N = 253)	Controls (N = 170)	*P^†^*
Age, years (mean ± SD)	59.7 ± 6.5	59.1 ± 5.5	0.518
Sex, female/male	129/124	87/83	0.735
BMI, kg/m˛ (mean ± SD)	30.1 ± 6.8	28.5 ± 7.2	0.073
Fasting glucose, mg/dL (median, IQR)	133, 115.2-163.8	90.9, 85.8-93.78	<0.001
Calcium, mg/dL	9.6 ± 0.3	9.5 ± 0.3	0.670
Phosphorus, mg/dL	3.6 ± 0.5	3.4 ± 0.3	0.090
25 (OH) D deficiency, n (%)	165 (65.4)	110 (64.7)	0.954
QTc interval, ms (median, IQR)	434, 418-467	423, 403-434	<0.001
QTc dispersion, ms (median, IQR)	53.8, 41-72.5	43.2, 31.9-53.9	<0.001

*SD – standard deviation; IQR – interquartile range; 25(OH)D – 25-hydroxyvitamin D; BMI – body mass index; QTc – corrected QT interval.

†*t* test or Mann-Whitney U test.

There was no significant difference in calcium (*P* = 0.670), phosphorus (*P* = 0.090), 25-OHD levels (*P* = 0.480), and the frequency of 25-OHD deficiency (*P* = 0.950) between the the diabetic group and controls. 25-OHD deficiency was found in 65.2% of patients and 64.7% of controls (*P* = 0.954). Men and women also did not significantly differ in the prevalence of 25-OHD deficiency (*P* = 0.721), QTc duration (*P* = 0.910), and QTc dispersion (*P* = 0.760).

Median QTc duration in patients with diabetes was 434 ms (IQR 418-467) and in controls 423 ms (IQR 403-434) (*P* < 0.001). A prolonged QTc interval was found in 26.8% (68 in 253) of patients and 4.1% (7 in 170) of controls (*P* < 0.001). In diabetic group, patients with prolonged QTc interval more frequently had 25-OHD deficiency than patients with normal QTc (*P* < 0.001). There were no significant differences between these groups in calcium and phosphorus levels (*P* = 0.114 and *P* = 0.752, respectively) (Table 2). In multivariate regression analysis the odds of prolonged QTc intervals increased significantly with age (*P* = 0.008), longer duration of diabetes (*P* = 0.012), higher HbA1c levels (*P* = 0.034), and vitamin D deficiency (*P* = 0.010) (Table 3). The model explained 80.9% of QTc duration. The presence of microalbuminuria (*P* = 0.347), neuropathy (*P* = 0.126), retinopathy (*P* = 0.310), and hypertension (*P* = 0.172) were similar in both groups.

**Table 2:** Laboratory data of diabetic patients according to their QTc interval*

	Prolonged QT interval (QT>440 ms) (N = 68)	Normal QT interval (QT<440 ms) (N = 185)	*P^†^*
Age, years (mean ± SD)	61.5 ± 6.6	59.0 ± 6.4	0.012
Sex, female/male	23/25	50/54	0.910
Duration of diabetes, years (median, IQR)	10, 5-17	8, 5-12	0.041
Fasting blood glucose, mg/dL (median, IQR)	136.2, 111.6-181.8	132.8, 110.3-169.2	0.195
HbA1c, % (mean ± SD)	7.4 ± 1.19	7.05 ± 1.09	0.011
Calcium, mg/dL (mean ± SD)	9.4 ± 0.3	9.6 ± 0.4	0.114
Phosphorus, mg/dL (mean ± SD)	3.7 ± 0.7	3.6 ± 0.4	0.752
25 (OH) D deficiency, n (%)	58 (85.2)	104 (56.2)	<0.001

*SD – standard deviation; IQR – interquartile range; 25(OH)D – 25-hydroxyvitamin D; HbA1c –hemoglobin A1c; QTc – corrected QT interval.

†*t* test or Mann-Whitney U test.

**Table 3:** Multivariate regression analysis for risk factors of prolonged QTc interval*

	95% confidence interval	Odds ratio	*P*
Age	1.03-1.25	1.14	0.008
Duration of diabetes	1.02-1.21	1.11	0.012
HbA1c	1.03-3.18	1.81	0.034
25 (OH) D deficiency	1.44-22.61	5.71	0.010

*25(OH)D – 25-hydroxyvitamin D; HbA1c – hemoglobin A1c; QTc – corrected QT interval.

The median QTc dispersion was 53.8 ms (IQR 41-72.5) in patients and 43.2 ms (IQR 31.9-53.9) in controls (*P* < 0.001). Prolonged QTc dispersion was detected in 20.9% of patients and 4.1% of controls (*P* < 0.001). Diabetic patients with prolonged QTc dispersion were of similar age and had similar HbA1c level and diabetes duration as patients with normal QTc dispersion. However, they significantly more frequently had 25-OHD deficiency (*P* = 0.010). There were no significant differences in calcium and phosphorus levels (*P* = 0.133 and *P* = 0.217 respectively), microalbuminuria (*P* = 0.173), neuropathy and retinopathy incidence (*P* = 0.376 and *P* = 0.832 respectively) between these groups (Table 4).

**Table 4:** Laboratory data of the patients according to QTc dispersion*

	Prolonged QTc dispersion (QTc >80 ms) (N = 53)	Normal QTc dispersion (QTc <80 ms) (N = 200)	*P^†^*
Age, years (mean ± SD)	60.5 ± 6.0	59.5 ± 6.7	0.182
Sex, female/male	18/19	56/60	0.764
Duration of diabetes, years (median, IQR)	9.5, 5-15.7	9, 5-12	0.600
Fasting blood glucose, mg/dL (median, IQR)	146.7, 109.8-183	140.7, 110.8-162.7	0.592
HbA1c (mean ± SD)	7.4 ± 1.1	7.0 ± 1.0	0.153
Calcium, mg/dL (mean ± SD)	9.4 ± 0.3	9.6 ± 0.3	0.133
Phosphorus, mg/dL (mean ± SD)	3.7 ± 0.7	3.6 ± 0.4	0.217
25 (OH) D deficiency, n (%)	43 (81.1)	112 (56)	0.010

*SD – standard deviation; IQR – interquartile range; 25(OH)D – 25-hydroxyvitamin D; HbA1c – hemoglobin A1c; QTc – corrected QT interval.

†*t* test or Mann-Whitney U test.

## Discussion

This study showed prolonged QTc interval and QTc dispersion in type 2 diabetic patients. Prolonged QTc interval was more frequent in older patients with higher HbA1c levels, longer diabetes duration, and 25-OHD deficiency. This is the first study that showed the association between QTc duration, dispersion, and 25-OHD deficiency in diabetic patients.

Although the best characterized sequels of 25-OHD deficiency affect the musculoskeletal system, the discovery that most tissues and many cells in the body have a vitamin D receptor has provided a new insight into the vitamin D metabolism. It was shown that the vitamin D axis affected vascular smooth muscle cell proliferation, inflammation, vascular calcification, renin angiotensin system, and blood pressure (14,15), all of which increase the risk of coronary vascular disease coronary vascular disease (CVD) and MI. In addition, vitamin D regulates calcium and phosphorus metabolism, and abnormalities in serum calcium concentrations affect QT interval duration (16).

QTc duration and QTc dispersion are the most frequently used parameters in the electrocardiographic assessment of ventricular repolarization. They have been associated with the risk of arrhythmogenesis. They have also been shown to be prolonged in diabetic patients (17,18). The prevalence of prolonged QTc has been reported to be as high as 16% in type 1 diabetes (19) and 30.1% in T2DM (20). The prevalence of prolonged QTc interval in our cohort was 26.8% and that of prolonged QTc dispersion was 20.9%. Another study found that QT interval in general population was inversely associated with serum calcium and positively associated with serum phosphorus, but there was no association with 25-OHD (21). In our diabetic cohort, prolonged QTc interval and dispersion were associated with vitamin 25-OHD deficiency, but not with calcium and phosphorus levels. Calcium plays an important role in determining the duration of the action potential of cardiac cells. It is well known that hypocalcemia prolongs QT interval due to longer phase 2 of the cardiac action potential (16). However in our study, patients with 25-OHD deficiency, although they had similar calcium levels as controls, had prolonged QTc interval. We may speculate that low 25-OHD levels may have an additional effect on the pathogenesis of prolonged QTc interval in patients with diabetes.

We also observed that patients with prolonged QTc interval had longer diabetes duration and higher HbA1c. Acute hyperglycemia has been shown to increase QT interval in newly diagnosed T2DM patients (22). The mechanisms involved in this process were increased intracellular calcium concentration and the impairment of sympatho-vagal balance (22). Additionally, hyperglycemia has been demonstrated to increase the sympathetic activity, which is evident as increased plasma cathecholamine production (23). In our cohort, prolonged QTc interval was also associated with age, but prolonged QTc dispersion was not. Mangoni et al (24) reported similar observations in healthy participants, explaining that they might be secondary to cardiac hypertrophy and myocardial action potential prolongation.

In our study, QTc duration and dispersion were linearly related. However, 34% of patients with normal QTc dispersion had a prolonged QTc interval. It was reported that QTc interval and QTc dispersion may reflect different aspects of abnormal ventricular repolarization (3). Our cohort comprised T2DM patients and in these patients several disease processes may influence myocardial depolarization and repolarization duration. Nevertheless, we found that 25-OHD deficiency prolonged both QTc interval and QTc dispersion.

25-OHD deficiency is highly prevalent in Turkey and worldwide (25,26). Its principal causes are limited cutaneous synthesis due to inadequate sun exposure or pigmented skin and cultural practices. The prevalence of 25-OHD deficiency in Turkey in the summer season is 60% (26), while the prevalence observed in our study, conducted in winter, was 65.2% in patients and 64.7% in controls. To avoid the effect of seasonal changes this study was performed between October and December.

The main limitation of this study is the manual assessment of the QTc interval and dispersion. However, superiority of available automatic methods has not been proven (27). Also, this study included patients who are receiving oral hypoglycemic therapy and without known cardiovascular disease, who are not representative of the overall type 2 diabetic population.

Despite these limitations, this study clearly demonstrated the relationship between 25-OHD deficiency and QTc parameters in diabetic patients. Assessment of 25-OHD levels in these patients may be important for better stratification of cardiac disease risk. However, these findings need to be confirmed by additional randomized trials as well as treatment studies.
